# Utilization of Parallel Resources and Sociodemographic Factors in Treating Children with Complex Chronic Diseases: A Cross-Sectional Study

**DOI:** 10.3390/children8110973

**Published:** 2021-10-28

**Authors:** Bibiana Pérez-Ardanaz, María José Peláez-Cantero, María González-Cano-Caballero, Laura Gutiérrez-Rodríguez, Alberto José Gómez-González, Inmaculada Lupiáñez-Pérez, José Miguel Morales-Asencio, José Carlos Canca-Sánchez

**Affiliations:** 1 Faculty of Health Sciences, Universidad de Málaga, 29071 Málaga, Spain; bibianap@uma.es (B.P.-A.); laura_gr@uma.es (L.G.-R.); gomezgonzalez88@uma.es (A.J.G.-G.); ilupianezperez@gmail.com (I.L.-P.); jccanca@uma.es (J.C.C.-S.); 2 Paediatric Palliative Care Unit, Hospital Regional Universitario de Málaga, 29011 Málaga, Spain; pelaez_mariajose@hotmail.com; 3 Faculty of Health Sciences, Universidad de Sevilla, 41009 Sevilla, Spain; mgonzalez79@us.es; 4 Instituto de Investigación Biomédica de Málaga (IBIMA), 29010 Málaga, Spain

**Keywords:** child health services, multiple chronic conditions, alternative therapies, social support, public health, psychosocial support systems, and socioeconomic factors

## Abstract

Background: Children with complex chronic conditions have a high need for health and social care resources. Many parents explore parallel resources such as alternative therapies, associations, psychological support, private medical consultations, and other out-of-pocket expenses for healthcare. The use of these alternative health resources is sometimes unclear and may lead to health inequalities. To characterize the use made of alternative healthcare resources for children with complex chronic conditions. Additionally, we evaluate the influence of sociodemographic factors on the distribution of this utilization of resources; (2) Methods: Cross-sectional study. Children with complex chronic diseases were treated at a tertiary hospital in Granada, Spain in 2016. We analyzed their use of healthcare resources and socioeconomic variables. This research complies with STROBE guidelines for observational studies; (3) Results: In total, 265 children were analyzed (mean age 7.3 years, SD 4.63). A total of 105 children (39.6%) attended private consultations with specialists, and 12.1% (*n* = 32) of the children had additional private health insurance. One out three parents belonged to a mutual support association (*n* = 78), and 26% (*n* = 69) of the children used alternative therapies. Furthermore, 75.4% (*n* = 199) of the children received no psychological support. Children whose parents had a higher educational level and occupations status made greater use of parallel healthcare resources.; (4) Conclusions: A significant proportion of children used multiple health resources in addition to the public healthcare system depending on sociodemographic determinants. Studies are needed to determine whether the use of these alternative services achieves better levels of health.

## 1. Introduction

Many children’s lives are threatened or limited by an increased prevalence of chronic diseases, which are characterized by major health-related needs, severe chronic conditions, functional limitations, and the frequent need for healthcare resources [1]. These circumstances have a substantial impact on the children and their families and pose a major challenge to health services, which have traditionally been oriented toward acute care. To address this challenge, health services need to be reoriented to enhanced care management and comprehensive person-centered care, aimed at improving both the quality and efficiency of healthcare for these children [2]. Despite the evidence provided in this respect and the recommendations made for the implementation of this type of care model [3], vulnerable children do not always have access to the necessary services, due to semi-permanent barriers affecting providers and parents alike [4]. Families often report that their greatest challenge is that of overcoming fragmented communication across systems, services, and providers [5], along with care continuity gaps and frustrations with access to health services [6], all of which generate unmet service needs, in association with family factors and the disease itself [7]. This issue is especially distressing in the case of children exposed to high levels of social vulnerability [8,9].

In those countries with a generalized public healthcare system, many parents explore parallel resources outside this setting. Thus, intending to seek the best options for the care and well-being of their children, they resort to optional resources such as alternative therapies (homeopathy, massage, reflexology, reiki, herbal medicines, aromatherapy, etc),), associations (non-profit patients and caregivers associations), psychological support (private psychology consultation), private medical consultations and other out-of-pocket expenses for healthcare [10]. However, healthcare staff may be unaware of these options and, moreover, they may not be compatible with the main service provided [11].

The use of these alternative health resources is sometimes unclear, and further study may clarify their potential impact on health inequalities and the care program provided. Moreover, a better understanding of the patterns of use of these resources could provide valuable information in developing a child and family-centered approach to healthcare.

The main aim of this study is to characterize the use made of alternative healthcare resources for children with complex chronic conditions. Additionally, we evaluate the influence of sociodemographic factors and the presence of life-threatening complex chronic conditions on the distribution of this utilization of resources.

## 2. Materials and Methods

### 2.1. Study Design

This study is cross-sectional and focused on children with complex chronic conditions. The study was designed in accordance with the conceptual framework for health inequalities developed by the Spanish Commission to Reduce Social Inequalities in Health among structural determinants of health inequalities (such as occupational status, social class, gender, education, psychosocial factors, and the healthcare system) [6]. Moreover, this study follows the Strengthening the Reporting of Observational Studies in Epidemiology (STROBE) Statement [12].

### 2.2. Participants

The study was conducted at the Virgen de las Nieves University Hospital in Granada (Spain), which is part of the national public healthcare system, and the reference hospital for the population of Granada aged under 18 years (170,808 inhabitants). In 2015, the hospital provided 66,382 pediatric consultations and treated 14,336 children, of whom 42 later died [13]. In subsequent years, mortality and population have yielded similar figures, being in 2019 167,214 inhabitants [14].

**Figure 1:**
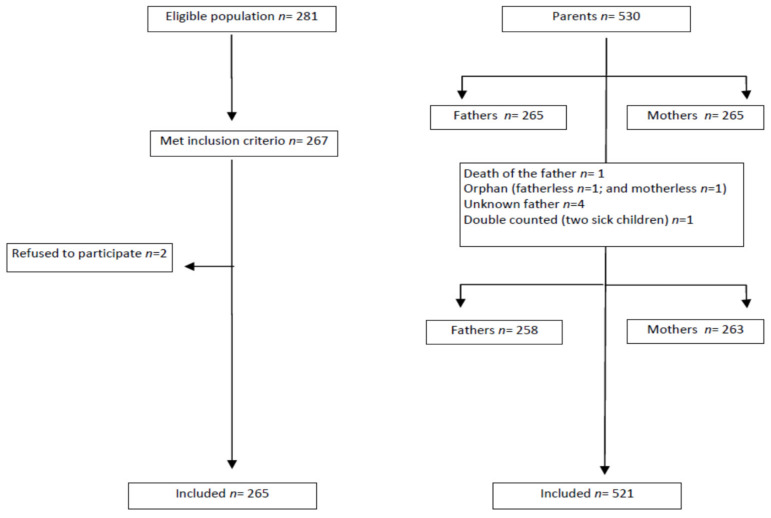
Participants flowchart.

The population considered for inclusion in the study were all children and adolescents aged under 18 years who had life-threatening complex chronic conditions (LT-CCCs) and were treated at the hospital in 2016. The children’s parents were approached during their visit to the hospital, and their consent to participate was requested, after ensuring that there was no life-threatening situation (expected death with poor prognosis in less than three months, severe immunosuppression status, or acute clinical instability), or any other condition that would limit understanding, verbal communication or the ability to provide consent. Assuming a prevalence of 10%, estimated from a previous pilot study carried out in the hospital, with a *p* = 0.05, with a precision of 6% and a 95% confidence level, 262 children were necessary to be recruited (Figure 1).

### 2.3. Data Collection

The participants were recruited from the lists of subjects attended during outpatient consultations, from hospitalized children or those referred by the case management nurse, after identification by their main diagnosis, according to the 9th International Classification of Diseases (ICD-9). Subsequently, children with LT-CCCs were identified according to the criteria proposed by Feudtner et al. [15], who classified nine organ system-based CCC types: cardiovascular, congenital/genetic, gastrointestinal, hematologic/immunologic, malignant, metabolic, neuromuscular, renal, and respiratory.

A database was made with the children cited and hospitalized with the inclusion criteria, reviewed daily by the principal researcher. Data were integrated into the database by standardized variables and pre-defined verification alerts to ensure uniform coding and to avoid errors during the population of the database.

After identifying the sample, the use of parallel health services (private health coverage, psychological support, mutual support association, private consultations, and alternative therapies) in the previous twelve months was ascertained from self-administered questionnaires delivered to the parents, together with the information about the study and request for signed informed consent. This process was conducted at the end of the consultation or during hospitalization, always in the presence of a member of the research team. The answers regarding utilization of health services were dichotomous (YES/NO). Private consultations were assumed for any visit to private medical specialists or any other type of additional private expenditures.

The questionnaire items included sociodemographic data such as participants’ age, gender, LT-CCCs. Social class (educational level and occupation) was measured on individual parents by neo-Weberian indicators of occupational social class (CSO-SEE12) [16].

### 2.4. Analysis

Descriptive and exploratory analyses were conducted to obtain frequencies, central and dispersion measures. The normality of distributions was evaluated by the Kolmogorov-Smirnov test. Bivariate analysis was performed to measure healthcare utilization by sociodemographic characteristics, LT-CCCs categories, or gender by chi-square test or Fisher’s test when indicated. Statistical significance for probability values was set at <0.05. Post-hoc power analyses were performed to evaluate the main bivariate analyses performed, such as the difference in private insurance by educational and occupational status. SPSS software (IBM Corp, Armonk, NY, USA). Version 24.0 was used for the statistical analysis.

## 3. Results

The sample was composed of 265 family groups (265 children, 265 fathers, and 265 mothers) (see participants flowchart-Figure 1). The fathers’ average age was 41.5 years (SD 7.8), and 38.6 years (SD 7.3) for mothers.

The father’s education profile showed how 40.3% (*n* = 104) had attained only a primary school level of education, and 36.1% (*n* = 92) were dedicated to an unskilled occupation. Among the mothers, the profile was similar, except that 29.7% (*n* = 78) had attained a university level of education vs. 22.1% (*n* = 57) for the fathers; 59.7% (*n* = 157) of the mothers were unemployed (Table 1).

The final sample of children consisted of 265 participants, ranging from 2 months to 17.3 years, with an average of 7.3 years (SD 4.6); 43.4% were female, 56.6% were male, and 89.4% (*n* = 237) were of Spanish nationality, and the rest from other countries. Children were treated mainly for neurological diseases (*n* = 87; 32.8%), congenital diseases (*n* = 60; 22.6%), oncological diseases (*n* = 46; 17.4%) and metabolic diseases (*n* = 37; 14%). Additionally, the complexity of children’s health status was evidenced by the number of them who needed medical devices at home such as continuous oxygen therapy (*n* = 41; 15.5%), mechanical ventilation (*n* = 7; 2.6%), enteral feeding (*n* = 29; 10.9%), urinary catheter (*n* = 8; 3.0%), gastrostomy (*n* = 36; 13.6%), or tracheostomy (*n* = 9; 3.4%).

### Healthcare Utilization and Sociodemographic Characteristics of the Parents

From the sample (Table 2), 12.1% (*n* = 32) of the parents had additional private health insurance. Most of these parents had a university degree (59.4% vs. 16.8% in fathers, χ^2^ = 30.9; *p* < 0.0001; 71.9% vs. 23.8% in mothers, χ^2^ = 31.9; *p* < 0.0001) or worked in a managerial or supervisory occupation (81.2% vs. 39.4% in fathers, χ^2^ = 37.1; *p* < 0.0001; 62.5% vs. 23.4% in mothers, χ^2^ = 34.3; *p* < 0.001). The post-hoc analyses revealed a power greater then 90% in all analyses.

Moreover, 40.3% (*n* = 104) attended private consultations with specialists or had other types of additional private expenses. This finding was significantly associated with education (66.3% vs. 46.7% in fathers, χ^2^ = 12.6; *p* = 0.006; 71.9% vs. 23.8% in mothers, χ^2^ = 31.9; *p* < 0.0001). A similar pattern was observed among the managerial/supervisory level of fathers (56.7% vs. 39.1, χ^2^ = 14.7; *p* = 0.002) and mothers (38.1% vs. 21.5%; χ^2^ = 10.1; *p* = 0.02). The post-hoc analyses revealed a power greater than 80% only in the case of mothers’ educational status, being lower in the rest of comparisons.

No psychological support was available for 75.4% (*n* = 199) of the parents. Psychological resources were accessed via associations (17.8%; *n* = 47), by private consultation (4.9%; *n* = 13), or by public hospital consultations (1.9%; *n* = 5). This support was more commonly received by children whose fathers (32.8% vs. 18.6%; χ^2^ = 10.2; *p* = 0.017) and mothers (46.2% vs. 24.2%; χ^2^ = 11.8; *p* = 0.008) had higher education and a high-level occupation (60.3% vs. 39.5%; χ^2^ = 15.9; *p* = 0.001 for fathers; 41.5% vs. 23.7%; χ^2^ = 8.8; *p* = 0.032 for mothers). The post-hoc analyses revealed a power under 80% in all these comparisons.

There was no difference among fathers regarding belonging to a mutual support association and having a higher educational level (29.3% vs. 18.7%; χ^2^ = 5.8; *p* = 0.118); although was significantly more frequent among the mothers with a university degree (40.3% vs. 25.5%; χ^2^ = 15.0; *p* = 0.002). With reference to occupation, those fathers with managerial employment (57.5% vs. 39.2%; χ^2^ = 10.7; *p* = 0.013), and mothers in the same situation (42.8% vs. 21.2%; χ^2^ = 15.2; *p* = 0.002), had more probability of belong to an organization for mutual support. The post-hoc power analyses yielded figures under 80% in all comparisons.

Fathers with university studies were most likely to use alternative therapies for their children (38.8% vs. 15.2% χ^2^ = 21.6; *p* < 0.0001), as were mothers (48.5% vs. 20.0%; χ^2^ = 26.4). Fathers with managerial and supervisory positions (65.1% vs. 42.3%; χ^2^ = 23.0; *p* < 0.0001) and mothers in analogous positions (41.1% vs. 22.5%; χ^2^ = 8.6; *p* = 0.034) made a greater use of alternative therapies. None of these analyses obtained a post-hoc power over 80%.

Analysis by categories showed that most of the spending on additional private consultations (48.6% vs. 22.5%; χ^2^ = 31.1; *p* < 0.001) was generated by children with neurological diseases (post-hoc power = 79%).

No significant differences were observed in the use of alternative therapies or mutual support associations by PCCC, while 50.8% vs. 6.5% of the spending on psychological support was for children with oncological diseases (50.8% vs. 6.5%; χ^2^ = 68.8; *p* < 0.0001) (post-hoc power = 94.1%).

Only four of those families that did not have Spanish nationality (14.3%) made use of private consultations (*p* = 0.004).

## 4. Discussion

The main aim of this study is to characterize the use of health resources additional to the services offered by the public health system, for children with complex chronic diseases, to identify patterns of use according to sociodemographic factors and LT-CCC criteria.

Our results highlight the existence of significant inequalities in the use of alternative health resources, in favor of children whose parents have better-paid jobs and higher education qualifications. Our findings corroborate Andersen’s description of the determinants of the use of health services, in his conceptual framework of inequalities [17], such as individual predisposing characteristics as parental educational level and enabling factors (occupation).

In Spain, health inequalities have been aggravated by the financial cutbacks imposed in response to the 2008 economic crisis, which has exacerbated differences in access to healthcare according to families’ ability to access additional services [18]. Moreover, this inequality is expected to increase in the coming years.

The Spanish health system is public, universal, and accessible to the entire population, although some restrictions were applied to the immigrant population during the economic crisis. Private health coverage, therefore, is an additional resource. Despite the risk of some needs remaining unmet by the public system, due to the above-mentioned financial cutbacks, rates of private health insurance are relatively low [19]. Although the preference for public health services remains stable [20], studies have shown that persons with higher incomes often have private health insurance, too [21], as is reflected in our own findings. However, in the case of preexisting disease, many insurers implement selective mechanisms to restrict the cover provided, particularly for populations with high levels of comorbidity. This could explain the lower percentage of private insurance obtained in our sample population, and the greater propensity to seek private consultations or specialist attention, paying the full amount of the service, a recourse that is not always possible for families with lower incomes [22]. Our results show there is a relationship between the use of health insurance and private consultations, and having a higher occupational and educational level, as previously detected by the Spanish National Health Survey for the general population [19], and literature review [23].

The low prevalence of complex chronic diseases among the child population may increase uncertainty about the disease prognosis. Moreover, this issue may be worsened by the absence of standardized treatments. Both of these factors are additional stressors that can heighten parents’ fear and anxiety [24,25] and encourage them to seek psychological support [26].

In addition to the above, in Spain, the provision of resources for the mental health of children and adolescents is irregular and often insufficient [27]. This fact, too, would contribute to the low accessibility (and hence use) of public-hospital psychological support.

Barely one-third of the parents in our sample belonged to a mutual support association related to their children’s illness. Feelings of isolation can have a stigmatizing effect and such mutual support associations can help develop awareness and provide information, thus providing parents with an additional resource (education programs) [28] to address the emotional, social, and informational challenges in caring for children with LT-CCCs [29]. However, associations do not exist for all diseases, and seeking them out, establishing contact, and remaining active can be difficult, and parents with greater financial and educational resources are often better equipped to identify and benefit from these associations to improve their children’s quality of life [30].

Our study shows that most use of alternative therapies is made by those parents with a higher occupational and educational level. There is a growing acceptance of these therapies among families of children and adolescents with multiple chronic conditions Nevertheless, in many cases, their effectiveness has been questioned [31], although there is growing evidence supporting the use of certain types like massage [32], art therapy [33], or animal-assisted therapy [34] for symptom management and to improve the quality of life. There are not many studies that have addressed the patterns of use of alternative therapies in children with chronic diseases versus healthy children, and they offer disparate results, with similar use in some studies and a higher rate in children with chronic conditions [35,36,37].

Our findings corroborate those of previous research in that the parents’ income and level of education are significant predictors of the recourse to alternative therapies [36,38]. Moreover, these therapies are most commonly employed for children with neurological diseases, which are also related to additional healthcare expenses [39,40].

This study is subject to certain limitations, such as a lack of statistical power to evaluate some comparisons by subgroups. Additionally, due to the cross-sectional design, no causal inference may be determined in the detected associations. Further research should be conducted to determine whether inequalities in the use of healthcare resources persist over time or whether our findings reflect a conjunctural finding. Furthermore, our study results may be strongly influenced by the characteristics of the Spanish model of public healthcare services; if so, they cannot readily be extrapolated to countries where there is no universal, free system of public healthcare insurance. Further, longitudinal, studies are needed to determine whether the use of these alternative services achieves better levels of health, in the medium and long term, among the population affected.

## 5. Conclusions

In Spain, unequal use is made of health services for children with complex chronic diseases. In many cases, is associated with the parents’ occupation and level of education. Although in some cases differences arise from the non-availability of certain services, in general, it is the parents with a higher occupational and educational level who are best able to identify and use alternative resources (although some, such as alternative therapies, are of unproven effectiveness).

These findings highlight the multifaceted nature of complexity. Parallel resources are not usually included in the dialogue between nurses and users when making decisions on children’s health care. Monitoring the use of different services and evaluating whether the care systems satisfy their needs considering socioeconomic inequalities, will assist in informing nursing services through identifying and allocating resources to reduce these barriers, and to offer comprehensive and coordinated care.

## Figures and Tables

**Table 1:** Characteristics of the sample.

Children’s	Male (*n* = 150)	Female (*n* = 115)		Parent’s	Male (*n* = 258)	Female (*n* = 263)	
	Mean (SD) or *n* (%)	Mean (SD) or *n* (%)	*p*		Mean (SD) or *n* (%)	Mean (SD) or *n* (%)	*p*
Age (years) †	7.39 (4.4)	7.18 (4.9)	0.714	Parents’ age †	44.37 (6.8)	41.52 (6.2)	<0.001
Health Recourses	Yes	None		Educational qualifications ‡			
Private Health coverage	32 (12.1)	233 (87.9)		None	13 (4.9)	11 (4.2)	<0.001
Psychological support	65 (24.5)	199 (75.1)		Primary	104 (40.3)	88 (33.5)	
Mutual support association	78 (29.4)	185 (69.8)		Secondary	28 (10.6)	26 (9.8)	
Private consultations	105 (39.6)	160 (60.4)		University	57 (22.1)	78 (29.7)	
Alternative therapies	69 (26.0)	161 (60.8)		Occupation ‡			
				Unemployed or retired	49 (19.2)	157 (59.7)	<0.001
				Managerial	36 (14.1)	37 (14.1)	
				Supervisory/intermediate level	78 (30.6)	37 (14.1)	
				Unskilled	92 (36.1)	32 (12.2)	

† Student’s *t*-test. ‡ Chi-square test.

**Table 2:** Health care utilization and sociodemographic characteristics.

	Father (*n* = 258)			Mother (*n* = 261)		
	Private Health Coverage					
	Public (*n* = 226)	Private (*n* = 32)		Public (*n* = 231)	Private (*n* = 32)	
	*n* (%)	*n* (%)	χ^2^	*n* (%)	*n* (%)	χ^2^
Parent’s education background						
-None	13 (5.8)	0 (0)	** 30.987	11 (4.8)	0 (0)	** 31.903
-Primary school	99 (43.8)	5 (15.6)		85 (36.8)	3 (9.4)	
-Secondary school	76 (33.6)	8 (25)		80 (34.6)	6 (18.8)	
-University	38 (16.8)	19 (59.4)		55 (23.8)	23 (71.9)	
Parent’s Professional occupation						
-Unemployed or retired	48 (21.5)	1 (3.1)	** 37.157	146 (63.2)	11 (34.4)	** 34.296
-Managers	21 (9.4)	15 (46.9)		22 (9.5)	15 (46.9)	
-Intermediate occupations	67 (30)	11 (34.4)		31 (13.4)	1 (3.1)	
-Unqualified occupation	87 (39)	5 (15.6)				
	Psychological support					
	Yes (*n* = 194)	None (*n* = 64)		Yes (*n* = 198)	None (*n* = 65)	
Parent’s education background						
-None	1 (1.6)	12 (6.2)	* 10.242	10 (5,1)	1 (1,5)	* 11.874
-Primary school	18 (28.1)	86 (44.3)		71 (35,9)	17 (26,2)	
-Secondary school	24 (37.5)	60 (30.9)		69 (34,8)	17 (26,2)	
-University	21 (32.8)	36 (18.6)		48 (24,2)	30 (46,2)	
Parent’s Professional occupation						
-Unemployed or retired	44 (22.9)	5 (7.9)	** 15.993	127 (64.1)	30 (46.2)	* 8.818
-Managers	19 (9.9)	17 (27)		25 (12.6)	12 (18.5)	
-Intermediate occupations	57 (29.7)	21 (33.3)		22 (11.1)	15 (23.1)	
-Unqualified occupation	72 (37.5)	20 (31.7)		24 (12.1)	8 (12.3)	
	Mutual support association					
	Yes (*n* = 75)	None (*n* = 183)		Yes (*n* = 77)	None (*n* = 186)	
Parent’s education background						
-None	3 (4)	10 (5.5)	5.870	5 (6.5)	6 (3.3)	* 15.052
-Primary school	23 (30.7)	81 (44.5)		13 (16.9)	75 (40.8)	
-Secondary school	27 (36)	57 (31.3)		28 (36.4)	56 (30.4)	
-University	22 (29.3)	34 (18.7)		31 (40.3)	47 (25.5)	
Parent’s Professional occupation						
-Unemployed or retired	9 (12.3)	40 (22.1)	* 10.760	37 (48,1)	120 (65.2)	* 15.260
-Managers	17 (23.3)	18 (9.9)		20 (26)	17 (9.2)	
-Intermediate occupations	25 (34.2)	53 (29.3)		13 (1.9)	22 (12)	
-Unqualified occupation	22 (30.1)	70 (38.7)		7 (9.1)	25 (13.6)	
	Private Consultations					
	Yes (*n* = 104)	None (*n* = 154)		Yes (*n* = 105)	None (*n* = 158)	
Parent’s education background						
-None	1 (1)	12 (7.8)	* 12.618	2 (1.9)	9 (5.7)	* 12.709
-Primary school	34 (32.7)	70 (45.5)		24 (22.9)	64 (40.5)	
-Secondary school	41 (39.4)	43 (27.9)		41 (39)	45 (28.5)	
-University	28 (26.9)	29 (18.8)		38 (36.2)	40 (25.3)	
Parent’s Professional occupation						
-Unemployed or retired	10 (9.6)	39 (25.8)	* 14.705	56 (53.3)	101 (63.9)	* 10.104
-Managers	20 (19.2)	16 (10.6)		22 (21)	15 (9.5)	
-Intermediate occupations	39 (37.5)	39 (25.8)		18 (17.1)	19 (12)	
-Unqualified occupation	35 (33.7)	57 (37.7)		9 (8.6)	23 (14.6)	
	Alternative therapies					
	Yes (*n* = 67)	None (*n* = 158)		Yes (*n* = 68)	None (*n* = 160)	
Parent’s education background						
-None	0 (0)	13 (8.2)	** 21.649	0 (0)	11 (6.9)	** 26.482
-Primary school	19 (28.4)	75 (47.5)		12 (17.6)	67 (41.9)	
-Secondary school	22 (32.8)	46 (29.1)		23 (33.8)	50 (31.3)	
-University	26 (38.8)	24 (15.2)		33 (48.5)	32 (20)	
Parent’s Professional occupation						
-Unemployed or retired	4 (6.1)	41 (26.3)	** 23.050	33 (48.5)	107 (66.9)	* 8.645
-Managers	18 (27.3)	14 (9)		15 (22.1)	18 (11.3)	
-Intermediate occupations	25 (37.9)	42 (26.9)		13 (19.1)	18 (11.3)	
-Unqualified occupation	19 (28.8)	59 (37.8)		7 (10.3)	17 (10.6)	

* Indicates a difference significant at the *p* ≤ 0.05 (** *p* ≤ 0.01) level of confidence.
